# Networked Fusion Filtering from Outputs with Stochastic Uncertainties and Correlated Random Transmission Delays

**DOI:** 10.3390/s16060847

**Published:** 2016-06-08

**Authors:** Raquel Caballero-Águila, Aurora Hermoso-Carazo, Josefa Linares-Pérez

**Affiliations:** 1 Departamento de Estadística, Universidad de Jaén, Campus Las Lagunillas, 23071 Jaén, Spain; 2 Departamento de Estadística, Universidad de Granada, Avda. Fuentenueva, 18071 Granada, Spain; ahermoso@ugr.es (A.H.-C.); jlinares@ugr.es (J.L.-P.)

**Keywords:** least-squares estimation, distributed and centralized fusion methods, random parameter matrices, correlated noises, random delays

## Abstract

This paper is concerned with the distributed and centralized fusion filtering problems in sensor networked systems with random one-step delays in transmissions. The delays are described by Bernoulli variables correlated at consecutive sampling times, with different characteristics at each sensor. The measured outputs are subject to uncertainties modeled by random parameter matrices, thus providing a unified framework to describe a wide variety of network-induced phenomena; moreover, the additive noises are assumed to be one-step autocorrelated and cross-correlated. Under these conditions, without requiring the knowledge of the signal evolution model, but using only the first and second order moments of the processes involved in the observation model, recursive algorithms for the optimal linear distributed and centralized filters under the least-squares criterion are derived by an innovation approach. Firstly, local estimators based on the measurements received from each sensor are obtained and, after that, the distributed fusion filter is generated as the least-squares matrix-weighted linear combination of the local estimators. Also, a recursive algorithm for the optimal linear centralized filter is proposed. In order to compare the estimators performance, recursive formulas for the error covariance matrices are derived in all the algorithms. The effects of the delays in the filters accuracy are analyzed in a numerical example which also illustrates how some usual network-induced uncertainties can be dealt with using the current observation model described by random matrices.

## 1. Introduction

Estimation problems in networked stochastic systems have been widely studied, especially in the past decade, due to the wide range of potential applications in many areas, such as target tracking, air traffic control, fault diagnosis, computer vision, and so on, and important advances in the design of multisensor fusion techniques have been achieved [1]. The basic question in the fusion estimation problems is how to merge the measurement data of different sensors; usually, the centralized and distributed fusion methods are used. Centralized fusion estimators are obtained by processing in the fusion center the measurements received from all sensors; in the distributed fusion method, first, local estimators, based on the measurements received from each sensor, are obtained and, then, these local estimators are combined according to a certain information fusion criterion. Several centralized and/or distributed fusion estimation algorithms have been proposed for conventional systems (see, e.g., [2,3,4,5], and the references therein), where the sensor measured outputs are affected only by additive noises, and assuming that the data are sent directly to the fusion center without any kind of transmission error; that is, assuming perfect connections.

However, although the use of sensor networks offers several advantages, due to restrictions of the physical equipment and uncertainties in the external environment, new problems associated with network-induced phenomena inevitably arise in both the output and the transmission of the sensor measurements [6]. Multiplicative noise uncertainties, sensor gain degradations and missing measurements are some of the random phenomena that usually arise in the sensor measured outputs, which cannot only be described by the usual additive disturbances. The existence of these uncertainties can severely degrade the performance of the conventional estimators, and has encouraged the need of designing new fusion estimation algorithms for such systems (see, e.g., [7,8,9,10,11], and references therein). Clearly, these situations with uncertainties in the measurements of the network sensors can be modeled by systems with random parameter measurement matrices, which have important practical significance due to the large number of realistic situations and application areas, such as digital control of chemical processes, radar control, navigation systems, or economic systems, in which this kind of systems with stochastic parameters are found (see, e.g., [12,13], among others). Therefore, it is not surprising that, in the past few years, much attention has been focused on the design of new fusion estimation algorithms for systems with random parameter matrices (see, e.g., [14,15,16,17], and references therein).

In addition to the aforementioned uncertainties, when the data packets are sent from the sensors to the processing center via a communication network, some further network-induced phenomena, such as random delays or measurement loss, inevitably arise during this transmission process, due to the unreliable network characteristics, imperfect communication channels and failures in the transmission. These additional transmission uncertainties can spoil the fusion estimators performance and motivate the need of designing fusion estimation algorithms that take their effects into consideration. In recent years, in response to the popularity of networked stochastic systems, the fusion estimation problem from observations with random delays and packet dropouts, which may happen during the data transmission, has been one of the mainstream research topics (see, e.g., [18,19,20,21,22,23,24,25,26,27,28,29,30], and references therein). All the above papers on signal estimation with random transmission delays assume independent random delays in each sensor and mutually independent delays between the different network sensors; in [31] this restriction was weakened and random delays featuring correlation at consecutive sampling times were considered, thus allowing to deal with common practical situations (e.g., those in which two consecutive observations cannot be delayed).

It should also be noted that in many real-world problems the measurement noises are usually correlated; for example, when all the sensors operate in the same noisy environment or when the sensor noises are state dependent. For this reason, the fairly conservative assumption that the measurement noises are uncorrelated is commonly weakened in most of the aforementioned research on signal estimation, including systems with both deterministic and random parameter matrices. Namely, the optimal Kalman filtering fusion problem in systems with cross-correlated noises at consecutive sampling times is addressed, for example, in [25]; also, under different types of noise correlation, centralized and distributed fusion algorithms are obtained in [11,26] for systems with multiplicative noise, and in [7] for systems where the measurements might have partial information about the signal. Autocorrelated and cross-correlated noises have been also considered in systems with random parameter matrices and transmission uncertainties; some results on the fusion estimation problems in these systems can be found in [22,24,27].

In this paper, covariance information is used to address the distributed and centralized fusion estimation problems for a class of linear networked stochastic systems with measured outputs perturbed by random parameter matrices, and subject to one-step random transmission delays. It is assumed that the sensor measurement noises are one-step autocorrelated and cross-correlated, and that the Bernoulli variables describing the measurement delays in the different sensors are correlated at the same and consecutive sampling times. The proposed observation model can describe the case of one-step delay, packet loss, or re-received measurements. To the best of the authors’ knowledge, fusion estimation problems in this framework with random measurement matrices and cross-correlated sensor noises in the measured outputs, together with correlated random delays in transmission, has not been investigated; so, encouraged by the above considerations, we reckon that it constitutes an interesting research challenge.

The main contributions of the current research are highlighted as follows: (i) The treatment used to address the estimation problem, based on covariance information, does not require the evolution model generating the signal process; nonetheless, the proposed fusion algorithms are also applicable to the conventional state-space model formulation; (ii) Random parameter matrices are considered in the measured outputs, which provide a fairly comprehensive and unified framework to describe some network-induced phenomena, such as multiplicative noise uncertainties or missing measurements, and correlation between the different sensor measurement noises is simultaneously considered; (iii) As in [31], random correlated delays in the transmission, with different delay characteristics at each sensor, are considered; however, the observation model in this paper is more general than that in [31], as the latter does not take random measurement matrices and correlated noises into account; (iv) Unlike [22,24,31], where only centralized fusion estimators are obtained, in this paper, both centralized and distributed estimation problems are addressed; the estimators are obtained under the innovation approach and recursive algorithms, very simple computationally and suitable for online applications, are proposed; (v) Finally, it must be noted that we do not use the augmented approach to deal with the delayed measurements, thus reducing the computational cost compared with the augmentation method.

The rest of the paper is organized as follows. Both, the measurement outputs with random parameter matrices, and the one-step random delay observation models are presented in Section 2, including the model hypotheses under which the distributed and centralized estimation problems are addressed. The distributed fusion method is applied in Section 3; specifically, the local least-squares linear filtering algorithms are derived in Section 3.1 using an innovation approach and, in Section 3.2, the proposed distributed fusion filter is obtained by a matrix-weighted linear combination of the local filtering estimators, using the mean squared error as optimality criterion. In Section 4, a recursive algorithm for the centralized least-squares linear filtering estimator is proposed. Section 5 is devoted to analyze the effectiveness of the proposed estimation algorithms by a simulation example, in which the effects of the sensor random delays on the estimators are compared. Some conclusions are drawn in Section 6.

**Notation:**  The notations throughout the paper are standard. The notation a∧b indicates the minimum value of two real numbers a,b. $R^{n}$ and $R^{m\times n}$ denote the *n*-dimensional Euclidean space and the set of all m×n real matrices, respectively. For a matrix *A*, the symbols $A^{T}$ and $A^{-1}$ denote its transpose and inverse, respectively; the notation A⊗B represents the Kronecker product of the matrices A,B. If the dimensions of vectors or matrices are not explicitly stated, they are assumed to be compatible with algebraic operations. In particular, *I* and 0 denote the identity matrix and the zero matrix of appropriate dimensions, respectively. For any function $G_{k,s}$, depending on the time instants *k* and *s*, we will write $G_{k}=G_{k,k}$ for simplicity; analogously, $F^{\left(i\right)}=F^{\left(ii\right)}$ will be written for any function $F^{\left(ij\right)}$, depending on the sensors *i* and *j*. Finally, $\delta_{k,s}$ denotes the Kronecker delta function.

## 2. Problem Formulation and Model Description

This paper deals with the distributed and centralized fusion filtering problems from randomly delayed observations coming from networked sensors; the signal measurements at the different sensors are noisy linear functions with random parameter matrices, and the sensor noises are assumed to be correlated and cross-correlated at the same and consecutive sampling times. The estimation is performed in a processing center, which is connected to all sensors, where the measurements are transmitted through unreliable communication channels which may lead to one-step random delays, due to network congestion or other causes. In the centralized filtering problem, the estimators are obtained by fusion of all the network observations at each sampling time, whereas in the distributed filtering, local estimators, based only on the observations of each individual sensor, are first obtained and then, a fusion estimator based on the local ones is calculated.

Our aim is to design recursive algorithms for the optimal linear distributed and centralized filters under the least-squares (LS) criterion, requiring only the first and second-order moments of the processes involved in the model describing the observations from the different sensors. Next, we present the model and formulate the hypotheses necessary to address the estimation problem.

*Description of the observation model.* Let us consider a discrete-time second-order $n_{x}$-dimensional signal process, $\left\{x_{k};\;k\geq 1\right\}$, which is measured in *m* sensor nodes of the network, i=1,…,m, that generate the outputs $z_{k}^{\left(i\right)}\in R^{n_{z}},\;k\geq 1$, described by

(1) $$z_{k}^{\left(i\right)}=H_{k}^{\left(i\right)}x_{k}+v_{k}^{\left(i\right)},\;\;k\geq 1;\;\;\;i=1,\ldots ,m$$

where $\left\{H_{k}^{\left(i\right)};\;k\geq 1\right\}$ are independent random parameter matrices of appropriate dimensions and $\left\{v_{k}^{\left(i\right)};\;k\geq 1\right\}$ is the process describing the measurement noise in sensor *i*, which is assumed to be one-step correlated. We also assume that all the sensors operate in the same noisy environment and there exists correlation between different sensor noises at the same and consecutive sampling times.

In order to estimate the signal process, $\left\{x_{k};\;k\geq 1\right\}$, the measurement outputs are transmitted to a processing center via unreliable channels, causing one-step random delays in such transmissions. When a measurement, $z_{k}^{\left(i\right)}$, suffers delay and, hence, it is unavailable at time *k*, the processor is assumed to use the previous one, $z_{k-1}^{\left(i\right)}$; this way of dealing with delays requires that the first measurement is always available and so, considering zero-one random variables $\gamma_{k}^{\left(i\right)},\;k\geq 2$, the observations used in the estimation are described by

(2) $$y_{k}^{\left(i\right)}=\left(1-\gamma_{k}^{\left(i\right)}\right)z_{k}^{\left(i\right)}+\gamma_{k}^{\left(i\right)}z_{k-1}^{\left(i\right)}\;,\;\;k\geq 2;\;\;\;y_{1}^{\left(i\right)}=z_{1}^{\left(i\right)};\;\;\;i=1,\ldots ,m$$

In this paper, the variables modelling the delays are assumed to be one-step correlated, thus covering many practical situations; for example those in which two consecutive observations through the same channel cannot be delayed, and situations where there is some sort of link between the different communications channels.

*Model hypotheses.* The LS estimation problem will be addressed under the following hypotheses about the processes involved in Equations (1) and (2), which formally specify the above assumptions:

*(H1)*  The signal process, $\left\{x_{k};\;k\geq 1\right\}$, has zero mean and its covariance function can be expressed in a separable form; namely, $E\left[x_{k}x_{s}^{T}\right]=A_{k}B_{s}^{T},\;s\leq k$, where $A_{k},B_{k},\;k\geq 1,$ are known matrices of dimensions $n_{x}\times N$.
*(H2)*  $\left\{H_{k}^{\left(i\right)};\;k\geq 1\right\}$, i=1,…,m, are independent sequences of independent random parameter matrices whose entries have known means and known second-order moments; we will denote ${\bar{H}}_{k}^{\left(i\right)}\equiv E\left[H_{k}^{\left(i\right)}\right],\;k\geq 1$.
*(H3)*  The sensor measurement noises $\left\{v_{k}^{\left(i\right)};\;k\geq 1\right\},\;i=1,\ldots ,m$, are zero-mean sequences with known second-order moments, defined by:
  $$E\left[v_{k}^{\left(i\right)}v_{s}^{(j)T}\right]=R_{k}^{\left(ij\right)}\delta_{k,s}+R_{k,k-1}^{\left(ij\right)}\delta_{k-1,s}\;,\;\;s\leq k;\;\;\;i,j=1,\ldots ,m$$
*(H4)*  $\left\{\gamma_{k}^{\left(i\right)};\;k\geq 2\right\},\;i=1,\ldots ,m,$ are sequences of Bernoulli random variables with known means, ${\bar{\gamma }}_{k}^{\left(i\right)}\equiv E\left[\gamma_{k}^{\left(i\right)}\right],\;k\geq 2$. It is assumed that $\gamma_{k}^{\left(i\right)}$ and $\gamma_{s}^{\left(j\right)}$ are independent for |k-s|≥2, and the second-order moments, ${\bar{\gamma }}_{k,s}^{\left(ij\right)}\equiv E\left[\gamma_{k}^{\left(i\right)}\gamma_{s}^{\left(j\right)}\right],\;s=k-1,k,$ and i,j=1,…,m, are also known.
*(H5)*  For i=1,…,m, the processes $\left\{x_{k};\;k\geq 1\right\},\;\left\{H_{k}^{\left(i\right)};\;k\geq 1\right\},\;\left\{v_{k}^{\left(i\right)};\;k\geq 1\right\}$ and $\left\{\gamma_{k}^{\left(i\right)};\;k\geq 2\right\}$ are mutually independent.

Hypotheses *(H1)–(H5)* guarantee that the observation processes in the different sensors have zero mean, and that the matrices $\Sigma_{k,s}^{y^{\left(ij\right)}}\equiv E\left[y_{k}^{\left(i\right)}y_{s}^{(j)T}\right],\;s=k-1,k$, can be obtained from $\Sigma_{k,s}^{z^{\left(ij\right)}}\equiv E\left[z_{k}^{\left(i\right)}z_{s}^{(j)T}\right],\;s=k-2,k-1,k$, and their transposes, by the following expressions:

(3) Σk,sy(ij)=1-γ¯k(i)-γ¯s(j)+γ¯k,s(ij)Σk,sz(ij)+γ¯s(j)-γ¯k,s(ij)Σk,s-1z(ij)+γ¯k(i)-γ¯k,s(ij)Σk-1,sz(ij)+Σk,sy(ij)=+γ¯k,s(ij)Σk-1,s-1z(ij),k,s≥2Σ2,1y(ij)=1-γ¯2(i)Σ2,1z(ij)+γ¯2(i)Σ1z(ij);Σ1y(ij)=Σ1z(ij)Σk,sz(ij)=EHk(i)AkBsTHs(j)T+Rk(ij)δk,s+Rk,k-1(ij)δk-1,s,s≤k

**Remark 1.**  *The term $E\left[H_{k}^{\left(i\right)}A_{k}B_{s}^{T}H_{s}^{(j)T}\right]$ in $\Sigma_{k,s}^{z^{\left(ij\right)}}$ becomes from $E\left[H_{k}^{\left(i\right)}x_{k}x_{s}^{T}H_{s}^{(j)T}\right]$ which, from the independence between $\left(x_{k},x_{s}\right)$ and $\left(H_{k}^{\left(i\right)},H_{s}^{\left(j\right)}\right)$, is equal to $E\left[H_{k}^{\left(i\right)}E\left[x_{k}x_{s}^{T}\right]H_{s}^{(j)T}\right]$. From* (H2)*, $E\left[H_{k}^{\left(i\right)}A_{k}B_{s}^{T}H_{s}^{(j)T}\right]={\bar{H}}_{k}^{\left(i\right)}A_{k}B_{s}^{T}{\bar{H}}_{s}^{(j)T}$ for j≠i or s≠k, and $E\left[H_{k}^{\left(i\right)}A_{k}B_{k}^{T}H_{k}^{(i)T}\right]$ is computed from its entries, according the following formula:*

$${\left(E\left[HA_{k}B_{k}^{T}H\right]\right)}_{\;pq}=\sum_{a=1}^{n_{x}}\sum_{b=1}^{n_{x}}E\left[h_{pa}h_{qb}\right]{\left(A_{k}B_{k}^{T}\right)}_{ab}\;,\;\;p,q=1,\ldots ,n_{z}\;$$

*where $H\in R^{n_{z}\times n_{x}}$ is any random matrix and $h_{pq}$ denote its entries.*

## 3. Distributed Fusion Linear Filter

The aim of this section is to address the distributed fusion linear filtering problem of the signal $x_{k}$, from the randomly delayed observations defined by Equations (1) and (2), under the LS criterion. The proposed distributed filter is designed as the LS matrix-weighted linear combination of the local LS linear filters and therefore, in a first step, such local filters need being derived.

### 3.1. Derivation of the Local LS Linear Filters

With the purpose of obtaining the signal LS linear filters based on the available observations from each sensor, we will use an innovation approach, which provides recursive algorithms for the local estimators, that will be denoted by ${\hat{x}}_{k/k}^{\left(i\right)}\;,\;i=1,\ldots ,m$.

For each sensor i=1,…,m, the innovation at time *k*, which represents the new information provided by the *k*-th observation, is defined by $\mu_{k}^{\left(i\right)}=y_{k}^{\left(i\right)}-{\hat{y}}_{k/k-1}^{\left(i\right)},\;k\geq 1$, where ${\hat{y}}_{k/k-1}^{\left(i\right)}$ is the LS linear estimator of $y_{k}$ based on $y_{s}^{\left(i\right)},\;s\leq k-1$, with ${\hat{y}}_{1/0}^{\left(i\right)}=E\left[y_{1}^{\left(i\right)}\right]=0$. As it is known [32], the innovations, $\left\{\mu_{k}^{\left(i\right)};\;k\geq 1\right\}$, constitute a zero-mean white process, and the LS linear estimator of any random vector $\xi_{k}$ based on the observations $y_{1}^{\left(i\right)},\ldots ,y_{L}^{\left(i\right)}$, denoted by ${\hat{\xi }}_{k/L}^{\left(i\right)}$, can be calculated as a linear combination of the corresponding innovations, $\mu_{1}^{\left(i\right)},\ldots \mu_{L}^{\left(i\right)}$; namely,

(4) $${\hat{\xi }}_{k/L}^{\left(i\right)}=\sum_{h=1}^{L}E\left[\xi_{k}\mu_{h}^{(i)T}\right]\Pi_{h}^{(i)-1}\mu_{h}^{\left(i\right)}$$

where $\Pi_{k}^{\left(i\right)}\equiv E\left[\mu_{k}^{\left(i\right)}\mu_{k}^{(i)T}\right]$ denotes the covariance matrix of $\mu_{k}^{\left(i\right)}$. This general expression is derived from the *Orthogonal Projection Lemma* (OPL), which establishes that the estimation error is uncorrelated with all the observations or, equivalently, uncorrelated with all the innovations.

Using the following alternative expression for the observations $y_{k}^{\left(i\right)}$ given by Equation (2),

(5) $$\begin{array}{c} y_{k}^{\left(i\right)}=\left(1-{\bar{\gamma }}_{k}^{\left(i\right)}\right)H_{k}^{\left(i\right)}x_{k}+{\bar{\gamma }}_{k}^{\left(i\right)}{\bar{H}}_{k-1}^{\left(i\right)}x_{k-1}+w_{k}^{\left(i\right)},\;\;k\geq 2\\ w_{k}^{\left(i\right)}={\bar{\gamma }}_{k}^{\left(i\right)}\left(H_{k-1}^{\left(i\right)}-{\bar{H}}_{k-1}^{\left(i\right)}\right)x_{k-1}+\left(1-{\bar{\gamma }}_{k}^{\left(i\right)}\right)v_{k}^{\left(i\right)}+{\bar{\gamma }}_{k}^{\left(i\right)}v_{k-1}^{\left(i\right)}-\left(\gamma_{k}^{\left(i\right)}-{\bar{\gamma }}_{k}^{\left(i\right)}\right)\left(z_{k}^{\left(i\right)}-z_{k-1}^{\left(i\right)}\right),\;\;k\geq 2 \end{array}$$

and taking into account the independence hypotheses stated on the model, it is easy to see, from Equation (4), that

(6) $${\hat{y}}_{k/k-1}^{\left(i\right)}=\left(1-{\bar{\gamma }}_{k}^{\left(i\right)}\right){\bar{H}}_{k}^{\left(i\right)}{\hat{x}}_{k/k-1}^{\left(i\right)}+{\bar{\gamma }}_{k}^{\left(i\right)}{\bar{H}}_{k-1}^{\left(i\right)}{\hat{x}}_{k-1/k-1}^{\left(i\right)}+\sum_{h=1}^{(k-1)\wedge 2}W_{k,k-h}^{\left(i\right)}\Pi_{k-h}^{(i)-1}\mu_{k-h}^{\left(i\right)}\;,\;\;k\geq 2$$

where $W_{k,k-h}^{\left(i\right)}\equiv E\left[w_{k}^{\left(i\right)}\mu_{k-h}^{(i)T}\right],\;h=1,2.$

The general Expression (4) for the LS linear estimators as linear combination of the innovations, together with Expression (6) for the one-stage observation predictor, are the starting point to derive the local recursive filtering algorithms presented below in Theorem 1; these algorithms provide also the filtering error covariance matrices, $P_{k/k}^{\left(i\right)}\equiv E\left[\left(x_{k}-{\hat{x}}_{k/k}^{\left(i\right)}\right){\left(x_{k}-{\hat{x}}_{k/k}^{\left(i\right)}\right)}^{T}\right]$, which measure the accuracy of the estimators ${\hat{x}}_{k/k}^{\left(i\right)}$ when the LS optimality criterion is used.

Hereafter, for the matrices $A_{k}$ and $B_{k}$ involved in the signal covariance factorization *(H1)*, the following operator will be used:

(7) $${\bar{H}}_{C_{k}}^{\left(i\right)}=\left(1-{\bar{\gamma }}_{k}^{\left(i\right)}\right){\bar{H}}_{k}^{\left(i\right)}C_{k}+{\bar{\gamma }}_{k}^{\left(i\right)}{\bar{H}}_{k-1}^{\left(i\right)}C_{k-1},\;\;k\geq 2;\;\;\;{\bar{H}}_{C_{1}}^{\left(i\right)}={\bar{H}}_{1}^{\left(i\right)}C_{1},\;\;\;C=A,B$$

**Theorem 1.**  *For each i=1,…,m, the local LS linear filters, ${\hat{x}}_{k/k}^{\left(i\right)},$ and the corresponding error covariance matrices, $P_{k/k}^{\left(i\right)}$, are given by*

(8) $$\begin{array}{c} {\hat{x}}_{k/k}^{\left(i\right)}=A_{k}O_{k}^{\left(i\right)},\;\;k\geq 1 \end{array}$$

*and*

(9) $$P_{k/k}^{\left(i\right)}=A_{k}{\left(B_{k}-A_{k}r_{k}^{\left(i\right)}\right)}^{T},\;\;k\geq 1$$

*where the vectors $O_{k}^{\left(i\right)}$ and the matrices $r_{k}^{\left(i\right)}=E\left[O_{k}^{\left(i\right)}O_{k}^{(i)T}\right]$ are recursively obtained from*

(10) $$O_{k}^{\left(i\right)}=O_{k-1}^{\left(i\right)}+J_{k}^{\left(i\right)}\Pi_{k}^{(i)-1}\mu_{k}^{\left(i\right)},\;\;k\geq 1;\;\;\;O_{0}^{\left(i\right)}=0$$

(11) $$r_{k}^{\left(i\right)}=r_{k-1}^{\left(i\right)}+J_{k}^{\left(i\right)}\Pi_{k}^{(i)-1}J_{k}^{(i)T},\;\;k\geq 1;\;\;r_{0}^{\left(i\right)}=0$$

*and the matrices $J_{k}^{\left(i\right)}=E\left[O_{k}^{\left(i\right)}\mu_{k}^{(i)T}\right]$ satisfy*

(12) $$J_{k}^{\left(i\right)}={\bar{H}}_{B_{k}}^{(i)T}-r_{k-1}^{\left(i\right)}{\bar{H}}_{A_{k}}^{(i)T}-\sum_{h=1}^{(k-1)\wedge 2}J_{k-h}^{\left(i\right)}\Pi_{k-h}^{(i)-1}W_{k,k-h}^{(i)T}\;,\;\;k\geq 2;\;\;\;J_{1}^{\left(i\right)}={\bar{H}}_{B_{1}}^{(i)T}$$

*The innovations $\mu_{k}^{\left(i\right)}$, and their covariance matrices, $\Pi_{k}^{\left(i\right)}$, are given by*

(13) $$\mu_{k}^{\left(i\right)}=y_{k}^{\left(i\right)}-{\bar{H}}_{A_{k}}^{\left(i\right)}O_{k-1}^{\left(i\right)}-\sum_{h=1}^{(k-1)\wedge 2}W_{k,k-h}^{\left(i\right)}\Pi_{k-h}^{(i)-1}\mu_{k-h}^{\left(i\right)}\;,\;\;k\geq 2;\;\;\;\mu_{1}^{\left(i\right)}=y_{1}^{\left(i\right)}$$

*and*

(14) $$\begin{array}{c} \Pi_{k}^{\left(i\right)}=\Sigma_{k}^{y^{\left(i\right)}}-{\bar{H}}_{A_{k}}^{\left(i\right)}\left({\bar{H}}_{B_{k}}^{(i)T}-J_{k}^{\left(i\right)}\right)-\sum_{h=1}^{(k-1)\wedge 2}W_{k,k-h}^{\left(i\right)}\Pi_{k-h}^{(i)-1}{\left({\bar{H}}_{A_{k}}^{\left(i\right)}J_{k-h}^{\left(i\right)}+W_{k,k-h}^{\left(i\right)}\right)}^{T},\;\;k\geq 2\\ \Pi_{1}^{\left(i\right)}=\Sigma_{1}^{y^{\left(i\right)}} \end{array}$$

*The coefficients $W_{k,k-h}^{\left(i\right)}=E\left[w_{k}^{\left(i\right)}\mu_{k-h}^{(i)T}\right]$, h=1,2, are calculated as*

(15) $$\begin{array}{c} W_{k,k-1}^{\left(i\right)}=\Sigma_{k,k-1}^{y^{\left(i\right)}}-{\bar{H}}_{A_{k}}^{\left(i\right)}{\bar{H}}_{B_{k-1}}^{(i)T}-W_{k,k-2}^{\left(i\right)}\Pi_{k-2}^{(i)-1}{\left({\bar{H}}_{A_{k-1}}^{\left(i\right)}J_{k-2}^{\left(i\right)}+W_{k-1,k-2}^{\left(i\right)}\right)}^{T},\;\;k\geq 3\\ W_{2,1}^{\left(i\right)}=\Sigma_{2,1}^{y^{\left(i\right)}}-{\bar{H}}_{A_{2}}^{\left(i\right)}{\bar{H}}_{B_{1}}^{(i)T} \end{array}$$

(16) $$\;W_{k,k-2}^{\left(i\right)}={\bar{\gamma }}_{k}^{\left(i\right)}\left(1-{\bar{\gamma }}_{k-2}^{\left(i\right)}\right)R_{k-1,k-2}^{\left(i\right)}\;,\;\;k\geq 4;\;\;\;W_{3,1}^{\left(i\right)}={\bar{\gamma }}_{3}^{\left(i\right)}R_{2,1}^{\left(i\right)}$$

Finally, the matrices $\Sigma_{k,s}^{y^{\left(i\right)}}$ and ${\bar{H}}_{A_{s}}^{\left(i\right)},\;{\bar{H}}_{B_{s}}^{\left(i\right)},\;s=k-1,k$, are given in Equations (3) and (7), respectively.

**Proof of Theorem 1.**  The local filter ${\hat{x}}_{k/k}^{\left(i\right)}$ will be obtained from the general Expression (4), starting from the computation of the coefficients $X_{k,h}^{\left(i\right)}=E\left[x_{k}\mu_{h}^{(i)T}\right]=E\left[x_{k}y_{h}^{(i)T}\right]-E\left[x_{k}{\hat{y}}_{h/h-1}^{(i)T}\right],\;1\leq h\leq k$.

The independence hypotheses and the separable structure of the signal covariance *(H1)* lead to $E\left[x_{k}y_{h}^{(i)T}\right]=A_{k}{\bar{H}}_{B_{h}}^{(i)T}$, with ${\bar{H}}_{B_{h}}^{\left(i\right)}$ given by Equation (7). From Expression (6) for ${\hat{y}}_{h/h-1}^{\left(i\right)},\;h\geq 2,$ we have:

$$E\left[x_{k}{\hat{y}}_{h/h-1}^{(i)T}\right]=\left(1-{\bar{\gamma }}_{h}^{\left(i\right)}\right)E\left[x_{k}{\hat{x}}_{h/h-1}^{(i)T}\right]{\bar{H}}_{h}^{(i)T}+{\bar{\gamma }}_{h}^{\left(i\right)}E\left[x_{k}{\hat{x}}_{h-1/h-1}^{(i)T}\right]{\bar{H}}_{h-1}^{(i)T}+\sum_{j=1}^{(h-1)\wedge 2}\;X_{k,h-j}^{\left(i\right)}\Pi_{h-j}^{(i)-1}W_{h,h-j}^{(i)T}$$

Hence, using now Equation (4) for ${\hat{x}}_{h/h-1}^{\left(i\right)}$ and ${\hat{x}}_{h-1/h-1}^{\left(i\right)}$, the filter coefficients are expressed as

Xk,h(i)=AkH¯Bh(i)T-∑j=1h-1Xk,j(i)Πj(i)-11-γ¯h(i)Xh,j(i)TH¯h(i)T+γ¯h(i)Xh-1,j(i)TH¯h-1(i)TXk,h(i)=-∑j=1(h-1)∧2Xk,h-j(i)Πh-j(i)-1Wh,h-j(i)T,2≤h≤k;Xk,1(i)=AkH¯B1(i)T

which guarantees that $X_{k,h}^{\left(i\right)}=A_{k}J_{h}^{\left(i\right)},\;1\leq h\leq k$, with $J_{h}^{\left(i\right)}$ given by

(17) $$J_{h}^{\left(i\right)}={\bar{H}}_{B_{h}}^{(i)T}-\sum_{j=1}^{h-1}J_{j}^{\left(i\right)}\Pi_{j}^{(i)-1}J_{j}^{(i)T}{\bar{H}}_{A_{h}}^{(i)T}-\;\;\sum_{j=1}^{(h-1)\wedge 2}J_{h-j}^{\left(i\right)}\Pi_{h-j}^{(i)-1}W_{h,h-j}^{(i)T}\;,\;h\geq 2;\;\;\;J_{1}^{\left(i\right)}={\bar{H}}_{B_{1}}^{(i)T}$$

Therefore, by defining $O_{k}^{\left(i\right)}=\sum_{h=1}^{k}J_{h}^{\left(i\right)}\Pi_{h}^{(i)-1}\mu_{h}^{\left(i\right)}$ and $r_{k}^{\left(i\right)}=E\left[O_{k}^{\left(i\right)}O_{k}^{(i)T}\right]$, Expression (8) for the filter follows immediately from Equation (4), and Equation (9) is obtained by using the OPL to express $P_{k/k}^{\left(i\right)}=E\left[x_{k}x_{k}^{T}\right]-E\left[{\hat{x}}_{k/k}^{\left(i\right)}{\hat{x}}_{k/k}^{(i)T}\right]$, and applying *(H1)* and Equation (8).

The recursive Expressions (10) and (11) are directly obtained from the corresponding definitions, taking into account that $r_{k}^{\left(i\right)}=\sum_{h=1}^{k}J_{h}^{\left(i\right)}\Pi_{h}^{(i)-1}J_{h}^{(i)T}$ which, in turn, from Equation (17), leads to Equation (12) for $J_{k}^{\left(i\right)}$.

From now on, using that ${\hat{x}}_{k/k-1}^{\left(i\right)}=A_{k}O_{k-1}^{\left(i\right)},\;{\hat{x}}_{k-1/k-1}^{\left(i\right)}=A_{k-1}O_{k-1}^{\left(i\right)}$ and Equation (7), Expression (6) for the observation predictor will be equivalently written as follows:

(18) $${\hat{y}}_{k/k-1}^{\left(i\right)}={\bar{H}}_{A_{k}}^{\left(i\right)}O_{k-1}^{\left(i\right)}+\sum_{h=1}^{(k-1)\wedge 2}W_{k,k-h}^{\left(i\right)}\Pi_{k-h}^{(i)-1}\mu_{k-h}^{\left(i\right)}\;,\;\;k\geq 2$$

From Equation (18), Expression (13) for the innovation is directly obtained and, applying the OPL to express its covariance matrix as $\Pi_{k}^{\left(i\right)}=E\left[y_{k}^{\left(i\right)}y_{k}^{(i)T}\right]-E\left[{\hat{y}}_{k/k-1}^{\left(i\right)}{\hat{y}}_{k/k-1}^{(i)T}\right]$, the following identity holds:

$$\Pi_{k}^{\left(i\right)}=\Sigma_{k}^{y^{\left(i\right)}}-{\bar{H}}_{A_{k}}^{\left(i\right)}E\left[O_{k-1}^{\left(i\right)}{\hat{y}}_{k/k-1}^{(i)T}\right]-\sum_{h=1}^{(k-1)\wedge 2}W_{k,k-h}^{\left(i\right)}\Pi_{k-h}^{(i)-1}E\left[\mu_{k-h}^{\left(i\right)}{\hat{y}}_{k/k-1}^{(i)T}\right],\;\;k\geq 2;\;\;\;\Pi_{1}^{\left(i\right)}=\Sigma_{1}^{y^{\left(i\right)}}$$

Now, using again Equation (18), it is deduced from Expression (12) that $E\left[O_{k-1}^{\left(i\right)}{\hat{y}}_{k/k-1}^{(i)T}\right]={\bar{H}}_{B_{k}}^{(i)T}-J_{k}^{\left(i\right)}$ and, since $E\left[{\hat{y}}_{k/k-1}^{\left(i\right)}\mu_{k-h}^{(i)T}\right]={\bar{H}}_{A_{k}}^{\left(i\right)}E\left[O_{k-1}^{\left(i\right)}\mu_{k-h}^{(i)T}\right]+W_{k,k-h}^{\left(i\right)}$ and $E\left[O_{k-1}^{\left(i\right)}\mu_{k-h}^{(i)T}\right]=J_{k-h}^{\left(i\right)}\;,\;h=1,2$, Expression (14) for $\Pi_{k}^{\left(i\right)}$ is obtained.

To complete the proof, the expressions for $W_{k,k-h}^{\left(i\right)}=E\left[w_{k}^{\left(i\right)}\mu_{k-h}^{(i)T}\right],\;h=1,2$, with $w_{k}^{\left(i\right)}$ given in Equation (5), are derived taking into account that $w_{k}^{\left(i\right)}$ is uncorrelated with $y_{h}^{\left(i\right)},\;h\leq k-3$. Consequently, $W_{k,k-2}^{\left(i\right)}=E\left[w_{k}^{\left(i\right)}y_{k-2}^{(i)T}\right]$, and Expression (16) is directly obtained from Equations (1), (2) and (5), using the hypotheses stated on the model. Next, using Equation (4) for ${\hat{y}}_{k-1/k-2}^{\left(i\right)}$ in $W_{k,k-1}^{\left(i\right)}=E\left[w_{k}^{\left(i\right)}y_{k-1}^{(i)T}\right]-E\left[w_{k}^{\left(i\right)}{\hat{y}}_{k-1/k-2}^{(i)T}\right]$, we have:

(19) $$W_{k,k-1}^{\left(i\right)}=E\left[w_{k}^{\left(i\right)}y_{k-1}^{(i)T}\right]-W_{k,k-2}^{\left(i\right)}\Pi_{k-2}^{(i)-1}{\left(E\left[y_{k-1}^{\left(i\right)}\mu_{k-2}^{(i)T}\right]\right)}^{T}$$

To compute the first expectation involved in this formula, we express $w_{k}^{\left(i\right)}=y_{k}^{\left(i\right)}-\left(1-{\bar{\gamma }}_{k}^{\left(i\right)}\right)H_{k}^{\left(i\right)}x_{k}-{\bar{\gamma }}_{k}^{\left(i\right)}H_{k-1}^{\left(i\right)}x_{k-1}$ and we apply the OPL to rewrite $E\left[x_{s}y_{k-1}^{(i)T}\right]=E\left[{\hat{x}}_{s/k-1}^{\left(i\right)}y_{k-1}^{(i)T}\right],\;s=k,k-1$, thus obtaining that $E\left[w_{k}^{\left(i\right)}y_{k-1}^{(i)T}\right]=\Sigma_{k,k-1}^{y^{\left(i\right)}}-{\bar{H}}_{A_{k}}^{\left(i\right)}E\left[O_{k-1}^{\left(i\right)}y_{k-1}^{(i)T}\right]$; then, by expressing $E\left[O_{k-1}^{\left(i\right)}y_{k-1}^{(i)T}\right]=E\left[O_{k-1}^{\left(i\right)}\mu_{k-1}^{(i)T}\right]+E\left[O_{k-1}^{\left(i\right)}{\hat{y}}_{k/k-1}^{(i)T}\right]$ and using Equations (12) and (18), it follows that $E\left[w_{k}^{\left(i\right)}y_{k-1}^{(i)T}\right]=\Sigma_{k,k-1}^{y^{\left(i\right)}}-{\bar{H}}_{A_{k}}^{\left(i\right)}{\bar{H}}_{B_{k-1}}^{(i)T}.$ The second expectation in Equation (19) is easily computed taking into account that, from the OPL, it is equal to $E\left[{\hat{y}}_{k-1/k-2}^{\left(i\right)}\mu_{k-2}^{(i)T}\right]$ and using Equation (18). So the proof is completed. ☐

### 3.2. Derivation of the Distributed LS Fusion Linear Filter

As it has been mentioned previously, a linear matrix-weighted fusion filter is now generated from the local filters by applying the LS optimality criterion. The distributed fusion filter at any time *k* is hence designed as a product, $F_{k}{\hat{X}}_{k/k}$, where ${\hat{X}}_{k/k}={\left({\hat{x}}_{k/k}^{(1)T},\ldots ,{\hat{x}}_{k/k}^{(m)T}\right)}^{T}$ is the vector constituted by the local filters, and $F_{k}\in R^{n_{x}\times mn_{x}}$ is a matrix such that the mean squared error, $E\left[{\left(x_{k}-F_{k}{\hat{X}}_{k/k}\right)}^{T}\left(x_{k}-F_{k}{\hat{X}}_{k/k}\right)\right]$, is minimized.

As it is known, the solution of this problem is given by $F_{k}^{opt}=E\left[x_{k}{\hat{X}}_{k/k}^{T}\right]{\left(E\left[{\hat{X}}_{k/k}{\hat{X}}_{k/k}^{T}\right]\right)}^{-1}$ and, consequently, the proposed distributed filter is expressed as:

(20) $${\hat{x}}_{k/k}^{\left(D\right)}=E\left[x_{k}{\hat{X}}_{k/k}^{T}\right]{\hat{\Sigma }}_{k/k}^{-1}{\hat{X}}_{k/k}$$

with ${\hat{\Sigma }}_{k/k}\equiv E\left[{\hat{X}}_{k/k}{\hat{X}}_{k/k}^{T}\right]={\left(E\left[{\hat{x}}_{k/k}^{\left(i\right)}{\hat{x}}_{k/k}^{(j)T}\right]\right)}_{i,j=1,\ldots ,m}$.

In view of Equation (20), and since the OPL guarantees that $E\left[x_{k}{\hat{X}}_{k/k}^{T}\right]=\left(E\left[{\hat{x}}_{k/k}^{\left(1\right)}{\hat{x}}_{k/k}^{(1)T}\right],\ldots ,E\left[{\hat{x}}_{k/k}^{\left(m\right)}{\hat{x}}_{k/k}^{(m)T}\right]\right)$, the derivation of ${\hat{x}}_{k/k}^{\left(D\right)}$ only requires the knowledge of the matrices ${\hat{\Sigma }}_{k/k}^{\left(ij\right)}\equiv E\left[{\hat{x}}_{k/k}^{\left(i\right)}{\hat{x}}_{k/k}^{(j)T}\right],\;i,j=1,\ldots ,m$.

The following theorem provides a recursive algorithm to compute the matrices ${\hat{\Sigma }}_{k/k}^{\left(ij\right)}$ which not only determine the proposed distributed fusion filter, but also the filtering error covariance matrix, $P_{k/k}^{\left(D\right)}=E\left[\left(x_{k}-{\hat{x}}_{k/k}^{\left(D\right)}\right){\left(x_{k}-{\hat{x}}_{k/k}^{\left(D\right)}\right)}^{T}\right]$.

**Theorem 2.**  *Let ${\hat{X}}_{k/k}={\left({\hat{x}}_{k/k}^{(1)T},\ldots ,{\hat{x}}_{k/k}^{(m)T}\right)}^{T}$ denote the vector constituted by the local LS filters given in Theorem 1, and ${\hat{\Sigma }}_{k/k}={\left({\hat{\Sigma }}_{k/k}^{\left(ij\right)}\right)}_{i,j=1,\ldots ,m}$ , with ${\hat{\Sigma }}_{k/k}^{\left(ij\right)}=E\left[{\hat{x}}_{k/k}^{\left(i\right)}{\hat{x}}_{k/k}^{(j)T}\right]$. Then, the distributed filtering estimator, ${\hat{x}}_{k/k}^{\left(D\right)}$, and the error covariance matrix, $P_{k/k}^{\left(D\right)}$, are given by*

(21) $${\hat{x}}_{k/k}^{\left(D\right)}=\left({\hat{\Sigma }}_{k/k}^{\left(1\right)}\;,\ldots ,{\hat{\Sigma }}_{k/k}^{\left(m\right)}\right){\hat{\Sigma }}_{k/k}^{-1}{\hat{X}}_{k/k}\;,\;\;k\geq 1$$

*and*

(22) $$P_{k/k}^{\left(D\right)}=A_{k}B_{k}^{T}-\left({\hat{\Sigma }}_{k/k}^{\left(1\right)}\;,\ldots ,{\hat{\Sigma }}_{k/k}^{\left(m\right)}\right){\hat{\Sigma }}_{k/k}^{-1}{\left({\hat{\Sigma }}_{k/k}^{\left(1\right)}\;,\ldots ,{\hat{\Sigma }}_{k/k}^{\left(m\right)}\right)}^{T},\;\;k\geq 1$$

*The matrices ${\hat{\Sigma }}_{k/k}^{\left(ij\right)}$, i,j=1,…,m, are computed by*

(23) $${\hat{\Sigma }}_{k/k}^{\left(ij\right)}=A_{k}r_{k}^{\left(ij\right)}A_{k}^{T},\;\;k\geq 1$$

*with $r_{k}^{\left(ij\right)}=E\left[O_{k}^{\left(i\right)}O_{k}^{(j)T}\right]$ satisfying*

(24) $$r_{k}^{\left(ij\right)}=r_{k-1}^{\left(ij\right)}+J_{k-1,k}^{\left(ij\right)}\Pi_{k}^{(j)-1}J_{k}^{(j)T}+J_{k}^{\left(i\right)}\Pi_{k}^{(i)-1}J_{k}^{(ji)T},\;\;k\geq 1;\;\;\;r_{0}^{\left(ij\right)}=0$$

*where $J_{k-1,k}^{\left(ij\right)}=E\left[O_{k-1}^{\left(i\right)}\mu_{k}^{(j)T}\right]$ are given by*

(25) $$\begin{array}{c} J_{k-1,k}^{\left(ij\right)}=\left(r_{k-1}^{\left(i\right)}-r_{k-1}^{\left(ij\right)}\right){\bar{H}}_{A_{k}}^{(j)T}+\sum_{h=1}^{(k-1)\wedge 2}J_{k-h}^{\left(i\right)}\Pi_{k-h}^{(i)-1}W_{k,k-h}^{(ji)T}-\sum_{h=1}^{(k-1)\wedge 2}J_{k-1,k-h}^{\left(ij\right)}\Pi_{k-h}^{(j)-1}W_{k,k-h}^{(j)T}\;,\;\;k\geq 2\\ J_{0,1}^{\left(ij\right)}=0 \end{array}$$

*and $J_{k,s}^{\left(ij\right)}=E\left[O_{k}^{\left(i\right)}\mu_{s}^{(j)T}\right],$ for s=k-1,k, satisfy*

(26) $$J_{k,s}^{\left(ij\right)}=J_{k-1,s}^{\left(ij\right)}+J_{k}^{\left(i\right)}\Pi_{k}^{(i)-1}\Pi_{k,s}^{\left(ij\right)},\;\;k\geq 2;\;\;\;J_{1}^{\left(ij\right)}=J_{1}^{\left(i\right)}\Pi_{1}^{(i)-1}\Pi_{1}^{\left(ij\right)}$$

*The innovation cross-covariance matrices $\Pi_{k}^{\left(ij\right)}=E\left[\mu_{k}^{\left(i\right)}\mu_{k}^{(j)T}\right]$ are obtained from*

(27) Πk(ij)=Σky(ij)-H¯Ak(i)H¯Bk(j)T-Jk(j)-Jk-1,k(ij)Πk(ij)=-∑h=1(k-1)∧2Wk,k-h(ij)Πk-h(j)-1H¯Ak(j)Jk-h(j)+Wk,k-h(j)T-∑h=1(k-1)∧2Wk,k-h(i)Πk-h(i)-1Πk-h,k(ij),k≥2Π1(ij)=Σ1y(ij)

*where $\Pi_{k,s}^{\left(ij\right)}=E\left[\mu_{k}^{\left(i\right)}\mu_{s}^{(j)T}\right],\;s=k-2,k-1$, are given by*

(28) $$\Pi_{k,s}^{\left(ij\right)}={\bar{H}}_{A_{k}}^{\left(i\right)}\left(J_{s}^{\left(j\right)}-J_{k-1,s}^{\left(ij\right)}\right)+W_{k,s}^{\left(ij\right)}-\sum_{h=1}^{(k-1)\wedge 2}\;W_{k,k-h}^{\left(i\right)}\Pi_{k-h}^{(i)-1}\Pi_{k-h,s}^{\left(ij\right)}\;,\;\;k\geq 2$$

*The coefficients $W_{k,k-h}^{\left(ij\right)}=E\left[w_{k}^{\left(i\right)}\mu_{k-h}^{(j)T}\right],\;h=1,2$, involved in the above expressions, are computed by*

(29) $$\begin{array}{c} W_{k,k-1}^{\left(ij\right)}=\Sigma_{k,k-1}^{y^{\left(ij\right)}}-{\bar{H}}_{A_{k}}^{\left(i\right)}{\bar{H}}_{B_{k-1}}^{(j)T}-W_{k,k-2}^{\left(ij\right)}\Pi_{k-2}^{(j)-1}{\left({\bar{H}}_{A_{k-1}}^{\left(j\right)}J_{k-2}^{\left(j\right)}+W_{k-1,k-2}^{\left(j\right)}\right)}^{T},\;\;k\geq 3\\ W_{3,2}^{\left(ij\right)}=\Sigma_{2,1}^{y^{\left(ij\right)}}-{\bar{H}}_{A_{2}}^{\left(i\right)}{\bar{H}}_{B_{1}}^{(j)T} \end{array}$$

(30) $$\;W_{k,k-2}^{\left(ij\right)}={\bar{\gamma }}_{k}^{\left(i\right)}\left(1-{\bar{\gamma }}_{k-2}^{\left(j\right)}\right)R_{k-1,k-2}^{\left(ij\right)}\;,\;\;k\geq 4;\;\;\;W_{3,1}^{\left(ij\right)}={\bar{\gamma }}_{3}^{\left(i\right)}R_{2,1}^{\left(ij\right)}$$

Finally, the matrices $\Sigma_{k,s}^{y^{\left(ij\right)}}$, and ${\bar{H}}_{A_{s}}^{\left(l\right)}\;,\;{\bar{H}}_{B_{s}}^{\left(l\right)},\;s=k-1,k,\;l=i,j$, are given in Equations (3) and (7), respectively.

**Proof.**  As it has been discussed previously, Expression (21) is immediately derived from Equation (20), while Equation (22) is obtained from $P_{k/k}^{\left(D\right)}=E\left[x_{k}x_{k}^{T}\right]-E\left[{\hat{x}}_{k/k}^{\left(D\right)}{\hat{x}}_{k/k}^{(D)T}\right]$, using *(H1)* and (21). Moreover, Equation (23) for ${\hat{\Sigma }}_{k/k}^{\left(ij\right)}$ is directly obtained using Equation (8) for the local filters and defining $r_{k}^{\left(ij\right)}=E\left[O_{k}^{\left(i\right)}O_{k}^{(j)T}\right]$.

Next, we derive the recursive formulas to obtain the matrices $r_{k}^{\left(ij\right)}$, which clearly satisfy Equation (24) by simply using Equation (10) and defining $J_{s,k}^{\left(ij\right)}=E\left[O_{s}^{\left(i\right)}\mu_{k}^{(j)T}\right],\;s=k-1,k$.

For subsequent derivations, the following expression of the one-stage predictor of $y_{k}^{\left(j\right)}$ based on the observations of sensor *i* will be used; this expression is obtained from Equation (5), taking into account, as proven in Theorem 1, that ${\hat{x}}_{k/s}^{\left(i\right)}=A_{k}O_{s}^{\left(i\right)},\;s=k-1,k$, and defining $W_{k,k-h}^{\left(ji\right)}=E\left[w_{k}^{\left(j\right)}\mu_{k-h}^{(i)T}\right],\;h=1,2$:

(31) $${\hat{y}}_{k/k-1}^{\left(j/i\right)}={\bar{H}}_{A_{k}}^{\left(j\right)}O_{k-1}^{\left(i\right)}+\sum_{h=1}^{(k-1)\wedge 2}W_{k,k-h}^{\left(ji\right)}\Pi_{k-h}^{(i)-1}\mu_{k-h}^{\left(i\right)}\;,\;\;k\geq 2$$

As Expression (31) is a generalization of Equation (18), hereafter we will also refer to it for the local predictors ${\hat{y}}_{k/k-1}^{\left(i\right)},\;k\geq 2$.

By applying the OPL, is clear that $E\left[O_{k-1}^{\left(i\right)}y_{k}^{(j)T}\right]=E\left[O_{k-1}^{\left(i\right)}{\hat{y}}_{k/k-1}^{(j/i)T}\right]$ and, consequently, we can rewrite $J_{k-1,k}^{\left(ij\right)}=E\left[O_{k-1}^{\left(i\right)}{\left({\hat{y}}_{k/k-1}^{\left(j/i\right)}-{\hat{y}}_{k/k-1}^{\left(j\right)}\right)}^{T}\right]$; then, using Equation (31) for both predictors, Expression (25) is easily obtained. Also, Equation (26) for $J_{k,s}^{\left(ij\right)},\;s=k-1,k$, is immediate from Equation (10), by simply defining $\Pi_{k,s}^{\left(ij\right)}=E\left[\mu_{k}^{\left(i\right)}\mu_{s}^{(j)T}\right]$.

To obtain Equation (27), firstly we apply the OPL to express $\Pi_{k}^{\left(ij\right)}=\Sigma_{k}^{y^{\left(ij\right)}}-E\left[{\hat{y}}_{k/k-1}^{\left(i/j\right)}{\hat{y}}_{k/k-1}^{(j)T}\right]-E\left[{\hat{y}}_{k/k-1}^{\left(i\right)}\mu_{k}^{(j)T}\right]$. Then, using Equation (31) for ${\hat{y}}_{k/k-1}^{\left(i/j\right)}$ and ${\hat{y}}_{k/k-1}^{\left(i\right)}\;$, and definitions of $J_{k-1,k}^{\left(ij\right)}$ and $\Pi_{k-h,k}^{\left(ij\right)}\;$, we have

$$\begin{array}{c} E\left[{\hat{y}}_{k/k-1}^{\left(i/j\right)}{\hat{y}}_{k/k-1}^{(j)T}\right]={\bar{H}}_{A_{k}}^{\left(i\right)}E\left[O_{k-1}^{\left(j\right)}{\hat{y}}_{k/k-1}^{(j)T}\right]+\sum_{h=1}^{(k-1)\wedge 2}W_{k,k-h}^{\left(ij\right)}\Pi_{k-h}^{(j)-1}E\left[\mu_{k-h}^{\left(j\right)}{\hat{y}}_{k/k-1}^{(j)T}\right],\\ E\left[{\hat{y}}_{k/k-1}^{\left(i\right)}\mu_{k}^{(j)T}\right]={\bar{H}}_{A_{k}}^{\left(i\right)}J_{k-1,k}^{\left(ij\right)}+\sum_{h=1}^{(k-1)\wedge 2}W_{k,k-h}^{\left(i\right)}\Pi_{k-h}^{(i)-1}\Pi_{k-h,k}^{\left(ij\right)}\; \end{array}$$

and Equation (27) is obtained taking into account that $E\left[O_{k-1}^{\left(j\right)}{\hat{y}}_{k/k-1}^{(j)T}\right]={\bar{H}}_{B_{k}}^{(j)T}-J_{k}^{\left(j\right)}$ and $E\left[{\hat{y}}_{k/k-1}^{\left(j\right)}\mu_{k-h}^{(j)T}\right]={\bar{H}}_{A_{k}}^{\left(j\right)}J_{k-h}^{\left(j\right)}+W_{k,k-h}^{\left(j\right)}\;$, as it has been derived in the proof of Theorem 1.

Next, Expression (28) for $\Pi_{k,s}^{\left(ij\right)}=E\left[y_{k}^{\left(i\right)}\mu_{s}^{(j)T}\right]-E\left[{\hat{y}}_{k/k-1}^{\left(i\right)}\mu_{s}^{(j)T}\right],$ with s=k-2,k-1, is obtained from $E\left[y_{k}^{\left(i\right)}\mu_{s}^{(j)T}\right]=H_{A_{k}}^{\left(i\right)}J_{s}^{\left(j\right)}+W_{k,s}^{\left(ij\right)}$, and using Equation (31) in $E\left[{\hat{y}}_{k/k-1}^{\left(i\right)}\mu_{s}^{(j)T}\right]$.

Finally, the reasoning for obtaining the coefficients $W_{k,k-h}^{\left(ij\right)}=E\left[w_{k}^{\left(i\right)}\mu_{k-h}^{(j)T}\right],\;h=1,2,$ is also similar to that of $W_{k,k-h}^{\left(i\right)}$ in Theorem 1, so it is omitted. Then the proof of Theorem 2 is completed. ☐

## 4. Centralized LS Fusion Linear Filter

In the centralized fusion filtering, the observations of the different sensors are jointly processed at each sampling time to yield the optimal filter of the signal $x_{k}$, which will be denoted by ${\hat{x}}_{k/k}^{\left(C\right)}$. To carry out this process, at each time k≥1 we will work with the vector constituted by the observations of all sensors, $y_{k}={\left(y_{k}^{(1)T},\ldots y_{k}^{(m)T}\right)}^{T}$, which, from Equation (2), can be expressed by

(32) $$y_{k}=\left(I-\Gamma_{k}\right)z_{k}+\Gamma_{k}z_{k-1},\;\;k\geq 2;\;\;\;y_{1}=z_{1}$$

where $z_{k}={\left(z_{k}^{(1)T},\ldots z_{k}^{(m)T}\right)}^{T}$ is the vector constituted by the sensor measured outputs given in Equation (1), and $\Gamma_{k}=Diag\left(\gamma_{k}^{\left(1\right)},\ldots ,\gamma_{k}^{\left(m\right)}\right)⊗I_{n_{z}}$. Let us note that, analogously to the sensor measured outputs $z_{k}^{\left(i\right)}$, the stacked vector $z_{k}$ is a noisy linear function of the signal, with random parameter matrices defined by $H_{k}={\left(H_{k}^{(1)T},\ldots H_{k}^{(m)T}\right)}^{T}$, and noise $v_{k}={\left(v_{k}^{(1)T},\ldots v_{k}^{(m)T}\right)}^{T}$:

(33) $$z_{k}=H_{k}x_{k}+v_{k}\;,\;\;k\geq 1$$

The processes involved in Equations (32) and (33) satisfy the following properties, which are immediately derived from the model hypotheses *(H1)–(H5)*:

*(P1)*  $\left\{H_{k};\;k\geq 1\right\}$ is a sequence of independent random parameter matrices whose entries have known means and second-order moments.
*(P2)*  The noise $\left\{v_{k};\;k\geq 1\right\}$ is a zero-mean sequence with known second-order moments defined by the matrices $R_{k,s}\equiv {\left(R_{k,s}^{\left(ij\right)}\right)}_{i,j=1,\ldots ,m}$.
*(P3)*  The matrices $\left\{\Gamma_{k};\;k\geq 2\right\}$ have known means, ${\bar{\Gamma }}_{k}\equiv E\left[\Gamma_{k}\right]=Diag\left({\bar{\gamma }}_{k}^{\left(1\right)},\ldots ,{\bar{\gamma }}_{k}^{\left(m\right)}\right)⊗I_{n_{z}}\;,\;k\geq 2$, and $\Gamma_{k}$ and $\Gamma_{s}$ are independent for |k-s|≥2.
*(P4)*  The processes $\left\{x_{k};\;k\geq 1\right\},\;\left\{H_{k};\;k\geq 1\right\},\;\left\{v_{k};\;k\geq 1\right\}$ and $\left\{\Gamma_{k};\;k\geq 2\right\}$ are mutually independent.

In view of Equations (32) and (33) and the above properties, the study of the LS linear filtering problem based on the stacked observations, $\left\{y_{k};\;k\geq 1\right\}$, is completely similar to that of the local filtering problem carried out in Section 3. Therefore, the centralized filtering algorithm described in the following theorem is derived by an analogous reasoning to that used in Theorem 1 and, hence, its proof is omitted.

**Theorem 3.**  *The centralized LS linear filter, ${\hat{x}}_{k/k}^{\left(C\right)},$ and the corresponding error covariance matrix, $P_{k/k}^{\left(C\right)}$, are given by*

$$\begin{array}{c} {\hat{x}}_{k/k}^{\left(C\right)}=A_{k}O_{k}\;,\;\;k\geq 1 \end{array}$$

*and*

$$P_{k/k}^{\left(C\right)}=A_{k}{\left(B_{k}-A_{k}r_{k}\right)}^{T},\;\;k\geq 1$$

*where the vectors $O_{k}$ and the matrices $r_{k}=E\left[O_{k}O_{k}^{T}\right]$ are recursively obtained from*

$$O_{k}=O_{k-1}+J_{k}\Pi_{k}^{-1}\mu_{k}\;,\;\;k\geq 1;\;\;\;O_{0}=0$$

$$r_{k}=r_{k-1}+J_{k}\Pi_{k}^{-1}J_{k}^{T},\;\;k\geq 1;\;\;\;r_{0}=0$$

*The matrices $J_{k}=E\left[O_{k}\mu_{k}^{T}\right]$ satisfy*

$$J_{k}={\bar{H}}_{B_{k}}^{T}-r_{k-1}{\bar{H}}_{A_{k}}^{T}-\sum_{h=1}^{(k-1)\wedge 2}J_{k-h}\Pi_{k-h}^{-1}W_{k,k-h}^{T}\;,\;\;k\geq 2;\;\;\;J_{1}={\bar{H}}_{B_{1}}^{T}$$

*The innovations, $\mu_{k}$, and their covariance matrices, $\Pi_{k}$, are given by*

$$\mu_{k}=y_{k}-{\bar{H}}_{A_{k}}O_{k-1}-\sum_{h=1}^{(k-1)\wedge 2}W_{k,k-h}\Pi_{k-h}^{-1}\mu_{k-h}\;,\;\;k\geq 2;\;\;\;\mu_{1}=y_{1}$$

*and*

$$\begin{array}{c} \Pi_{k}=\Sigma_{k}^{y}-{\bar{H}}_{A_{k}}\left({\bar{H}}_{B_{k}}^{T}-J_{k}\right)-\sum_{h=1}^{(k-1)\wedge 2}W_{k,k-h}\Pi_{k-h}^{-1}{\left({\bar{H}}_{A_{k}}J_{k-h}+W_{k,k-h}\right)}^{T},\;\;k\geq 2;\;\;\;\;\Pi_{1}=\Sigma_{1}^{y} \end{array}$$

*and the coefficients $W_{k,k-h}=E\left[w_{k}\mu_{k-h}^{T}\right],$h=1,2, verify*

$$\begin{array}{c} W_{k,k-1}=\Sigma_{k,k-1}^{y}-{\bar{H}}_{A_{k}}{\bar{H}}_{B_{k-1}}^{T}-W_{k,k-2}\Pi_{k-2}^{-1}{\left({\bar{H}}_{A_{k-1}}J_{k-2}+W_{k-1,k-2}\right)}^{T},\;\;k\geq 3\\ W_{2,1}=\Sigma_{2,1}^{y}-{\bar{H}}_{A_{2}}{\bar{H}}_{B_{1}}^{T} \end{array}$$

$$\;W_{k,k-2}={\bar{\Gamma }}_{k}R_{k-1,k-2}\left(I-{\bar{\Gamma }}_{k-2}\right),\;\;k\geq 4;\;\;\;W_{3,1}={\bar{\Gamma }}_{3}R_{2,1}$$

In the above formulas, the matrices $\Sigma_{k}^{y}$ and $\Sigma_{k,k-1}^{y}$ are computed by $\Sigma_{k,s}^{y}={\left(\Sigma_{k,s}^{y^{\left(ij\right)}}\right)}_{i,j=1,\ldots ,m}\;,\;s=k,k-1$, with $\Sigma_{k,s}^{y^{\left(ij\right)}}$ given in Equation (3), and ${\bar{H}}_{C_{k}}={\left({\bar{H}}_{C_{k}}^{(1)T},\ldots ,{\bar{H}}_{C_{k}}^{(m)T}\right)}^{T},\;C=A,B$, with ${\bar{H}}_{C_{k}}^{\left(i\right)}$ defined in Equation (7).

## 5. Numerical Simulation Example

This section is devoted to analyze the effectiveness of the proposed distributed and centralized filtering algorithms by a simulation example. Let us consider a zero-mean scalar signal process, $\left\{x_{k};\;k\geq 1\right\}$, with autocovariance function $E\left[x_{k}x_{s}\right]=1.025641\times 0.95^{k-s},\;\;s\leq k,$ which is factorizable according to *(H1)* just taking, for example, $A_{k}=1.025641\times 0.95^{k}$ and $B_{k}=0.95^{-k}.$

The measured outputs of this signal, which are provided by four different sensors, are described by Equation (1):

$$z_{k}^{\left(i\right)}=H_{k}^{\left(i\right)}x_{k}+v_{k}^{\left(i\right)},\;k\geq 1,\;i=1,2,3,4$$

where the processes $\left\{H_{k}^{\left(i\right)};\;k\geq 1\right\}$ and $\left\{v_{k}^{\left(i\right)};\;k\geq 1\right\}$, i=1,2,3,4, are defined as follows:

- $H_{k}^{\left(1\right)}=0.8\lambda_{k}^{\left(1\right)}$, $H_{k}^{\left(2\right)}=\lambda_{k}^{\left(2\right)}\left(0.75+0.95\varepsilon_{k}\right)$, $H_{k}^{\left(3\right)}=0.8\lambda_{k}^{\left(3\right)}$ and $H_{k}^{\left(4\right)}=0.75\lambda_{k}^{\left(4\right)}$, where $\left\{\varepsilon_{k};\;k\geq 1\right\}$ is a zero-mean Gaussian white process with unit variance, and $\left\{\lambda_{k}^{\left(i\right)};\;k\geq 1\right\}$, i=1,2,3,4, are white processes with the following time-invariant probability distributions:
  – For i=1,2, $\lambda_{k}^{\left(i\right)}$ are Bernoulli random variables with $P[\lambda_{k}^{\left(i\right)}=1]=0.6$.
  – $\lambda_{k}^{\left(3\right)}$ is uniformly distributed over [0.1,0.9].
  – $P\left[\lambda_{k}^{\left(4\right)}=0\right]=0.3,\;\;P\left[\lambda_{k}^{\left(4\right)}=0.5\right]=0.3,\;\;P\left[\lambda_{k}^{\left(4\right)}=1\right]=0.4.$
  It is also assumed that the sequences $\left\{\varepsilon_{k};\;k\geq 1\right\}$ and $\{\lambda_{k}^{\left(i\right)};\;k\geq 1\},\;i=1,2,3,4,$ are mutually independent.
- The additive noises are defined as $v_{k}^{\left(i\right)}=c_{i}\left(\eta_{k}+\eta_{k+1}\right),\;i=1,2,3,4$, where $c_{1}=c_{4}=0.75$, $c_{2}=1$, $c_{3}=0.5$, and $\left\{\eta_{k};\;k\geq 1\right\}$ is a zero-mean Gaussian white process with unit variance.

Note that the sequences of random variables $\left\{H_{k}^{\left(i\right)};\;k\geq 1\right\}$, i=1,2,3,4, model different types of uncertainty in the measured outputs: *missing measurements* in sensor 1; both *missing measurements and multiplicative noise* in sensor 2; and *continuous and discrete gain degradation* in sensors 3 and 4, respectively. Moreover, it is clear that the additive noises $\left\{v_{k}^{\left(i\right)};\;k\geq 1\right\}$, i=1,2,3,4, are only correlated at the same and consecutive time instants, with $R_{k}^{\left(ij\right)}=2c_{i}c_{j}\;,\;R_{k,k-1}^{\left(ij\right)}=c_{i}c_{j}\;,\;i,j=1,2,3,4.$

Next, according to our theoretical observation model, it is supposed that, at any sampling time k≥2, the data transmissions are subject to random one-step delays with different rates and such delays are correlated at consecutive sampling times. More precisely, let us assume that the available measurements $y_{k}^{\left(i\right)}$ are given by Equation (2):

$$y_{k}^{\left(i\right)}=\left(1-\gamma_{k}^{\left(i\right)}\right)z_{k}^{\left(i\right)}+\gamma_{k}^{\left(i\right)}z_{k-1}^{\left(i\right)}\;,\;\;k\geq 2,\;i=1,2,3,4$$

where the variables $\gamma_{k}^{\left(i\right)}$ modeling this type of correlated random delays are defined using three independent sequences of independent Bernoulli random variables, $\left\{\theta_{k}^{\left(i\right)};\;k\geq 1\right\}$, i=1,2,3, with constant probabilities, $P\left[\theta_{k}^{\left(i\right)}=1\right]={\bar{\theta }}^{\left(i\right)}$, for all k≥1; specifically, for i=1,2,3, $\gamma_{k}^{\left(i\right)}=\theta_{k+1}^{\left(i\right)}\left(1-\theta_{k}^{\left(i\right)}\right),$ and $\gamma_{k}^{\left(4\right)}=\theta_{k}^{\left(1\right)}\left(1-\theta_{k+1}^{\left(1\right)}\right).$

It is clear that the sensor delay probabilities are time-invariant: ${\bar{\gamma }}^{\left(i\right)}={\bar{\theta }}^{\left(i\right)}\left(1-{\bar{\theta }}^{\left(i\right)}\right)$, for i=1,2,3, and ${\bar{\gamma }}^{\left(4\right)}={\bar{\gamma }}^{\left(1\right)}$. Moreover, the independence of the sequences $\left\{\theta_{k}^{\left(i\right)}\;;\;k\geq 1\right\}$, i=1,2,3, together with the independence of the variables in each sequence, guarantee that the random variables $\gamma_{k}^{\left(i\right)}$ and $\gamma_{s}^{\left(j\right)}$ are independent if |k-s|≥2, for any i,j=1,2,3,4. Also, it is clear that, at each sensor, the variables $\left\{\gamma_{k}^{\left(i\right)}\;;\;k\geq 2\right\}$ are correlated at consecutive sampling times and ${\bar{\gamma }}_{k,s}^{\left(ii\right)}=0$, for i=1,2,3,4 and |k-s|=1. Finally, we have that $\left\{\gamma_{k}^{\left(4\right)};\;k\geq 2\right\}$ is independent of $\left\{\gamma_{k}^{\left(i\right)}\;;\;k\geq 2\right\}$, i=2,3, but correlated with $\left\{\gamma_{k}^{\left(1\right)}\;;\;k\geq 2\right\}$ at consecutive sampling times, with ${\bar{\gamma }}_{k,k-1}^{\left(14\right)}={\bar{\gamma }}^{\left(1\right)}{\bar{\theta }}^{\left(1\right)}$ and ${\bar{\gamma }}_{k,k-1}^{\left(41\right)}={\bar{\gamma }}^{\left(1\right)}\left(1-{\bar{\theta }}^{\left(1\right)}\right)$.

Let us observe that, for each sensor i=1,2,3,4, if $\gamma_{k}^{\left(i\right)}=1$, then $\gamma_{k+1}^{\left(i\right)}=0$; this fact guarantees that, when the measurement at time *k* is delayed, the available measurement at time k+1 is well-timed. Therefore, this correlation model avoids the possibility of two consecutive delayed observations at the same sensor. Table 1 shows an example of data transmission in sensor 1 when ${\bar{\theta }}^{\left(1\right)}=0.5$.

Note that if $\gamma_{k}^{\left(1\right)}=0$, there is no delay at time *k*; *i.e.*, the output $z_{k}^{\left(1\right)}$ is received on time by the fusion center and $y_{k}^{\left(1\right)}=z_{k}^{\left(1\right)}$. If $\gamma_{k}^{\left(1\right)}=1$, the packet received at time *k* is the output at k-1; *i.e.*, there is one-step delay and the processed observation is $y_{k}^{\left(1\right)}=z_{k-1}^{\left(1\right)}$. From Table 1, we see that $z_{4}^{\left(1\right)},\;z_{7}^{\left(1\right)}$ and $z_{11}^{\left(1\right)}$ are are lost, and $z_{3}^{\left(1\right)},\;z_{6}^{\left(1\right)}$ and $z_{10}^{\left(1\right)}$ are re-received.

To illustrate the feasibility and analyze the effectiveness of the proposed estimators, the algorithms were implemented in MATLAB, and a hundred iterations were run. In order to measure the estimation accuracy, the error variances of both distributed and centralized fusion estimators were calculated for several values of the delay probabilities at the different sensors, obtained from several values of ${\bar{\theta }}^{\left(i\right)}$. Let us observe that the delay probabilities, ${\bar{\gamma }}^{\left(i\right)}={\bar{\theta }}^{\left(i\right)}\left(1-{\bar{\theta }}^{\left(i\right)}\right)$, for i=1,2,3, are the same if $1-{\bar{\theta }}^{\left(i\right)}$ is used instead of ${\bar{\theta }}^{\left(i\right)}$; for this reason, only the case ${\bar{\theta }}^{\left(i\right)}\leq 0.5$ will be considered here.

**Figure 1:**
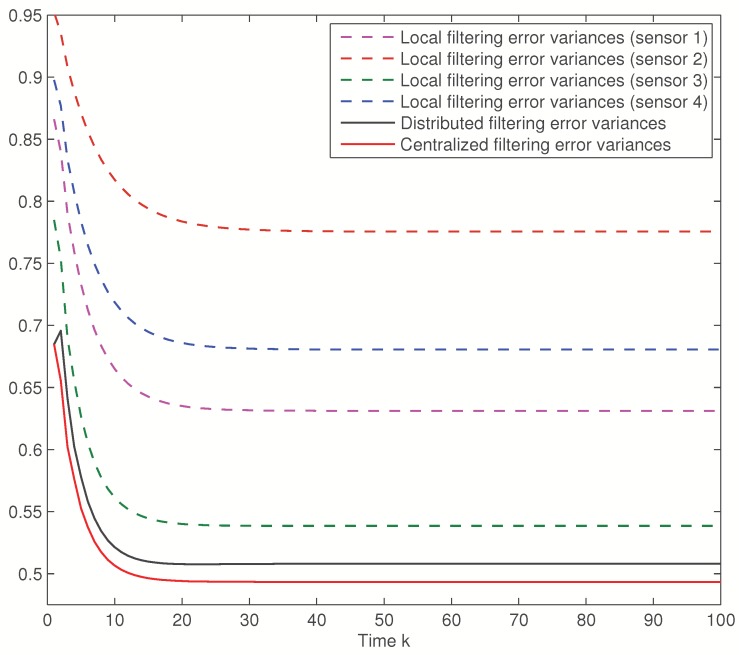
Local, distributed and centralized filtering error variances for ${\bar{\theta }}^{\left(i\right)}=0.3$, i=1,2,3.

**Figure 2:**
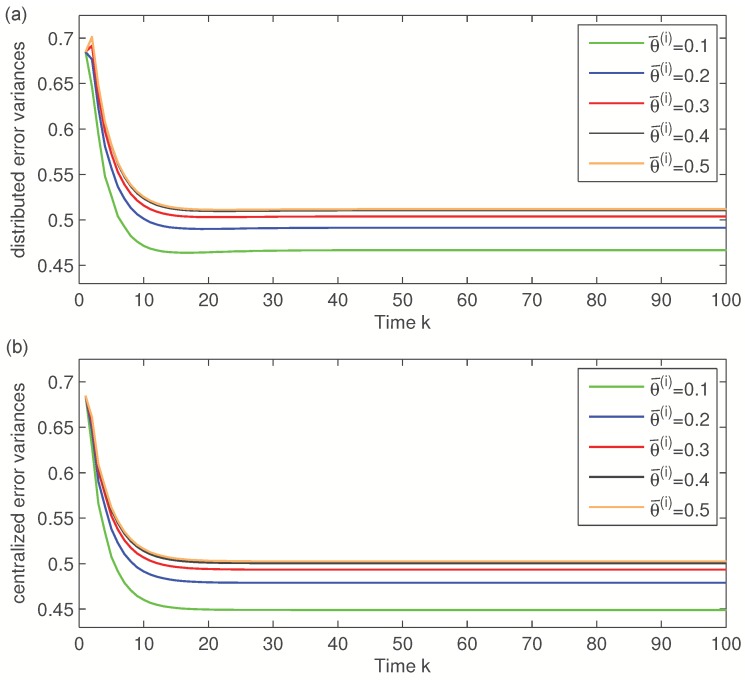
(**a**) Distributed filtering error variances and (**b**) centralized filtering error variances for different values of ${\bar{\theta }}^{\left(i\right)}$.

First, the error variances of the local, distributed and centralized filters will be compared considering the same delay probabilities for the four sensors. Figure 1 displays these error variances when ${\bar{\theta }}^{\left(i\right)}=0.3$, i=1,2,3, which means that the delay probability at every sensor is ${\bar{\gamma }}^{\left(i\right)}=0.21$. This figure shows that the error variances of the distributed fusion filtering estimator are lower than those of every local estimators, but slightly greater than those of the centralized one. However, this slight difference is compensated by the fact that the distributed fusion structure reduces the computational cost and has better robustness and fault tolerance. Analogous results are obtained for other values of ${\bar{\theta }}^{\left(i\right)}$. Actually, Figure 2 displays the distributed and centralized filtering error variances for ${\bar{\theta }}^{\left(i\right)}=0.1,\;0.2,\;0.3,\;0.4,\;0.5$, which lead to the delay probabilities ${\bar{\gamma }}^{\left(i\right)}=0.09,\;0.16,\;0.21,\;0.24,\;0.25$, respectively. In this figure, both graphs (corresponding to the distributed and centralized fusion filters, respectively) show that the performance of the filters is poorer as ${\bar{\theta }}^{\left(i\right)}$ increases. This fact was expected as the increase of ${\bar{\theta }}^{\left(i\right)}$ yields a rise in the delay probabilities. This figure also confirms that both methods, distributed and centralized, have approximately the same accuracy, thus corroborating the previous results.

**Figure 3:**
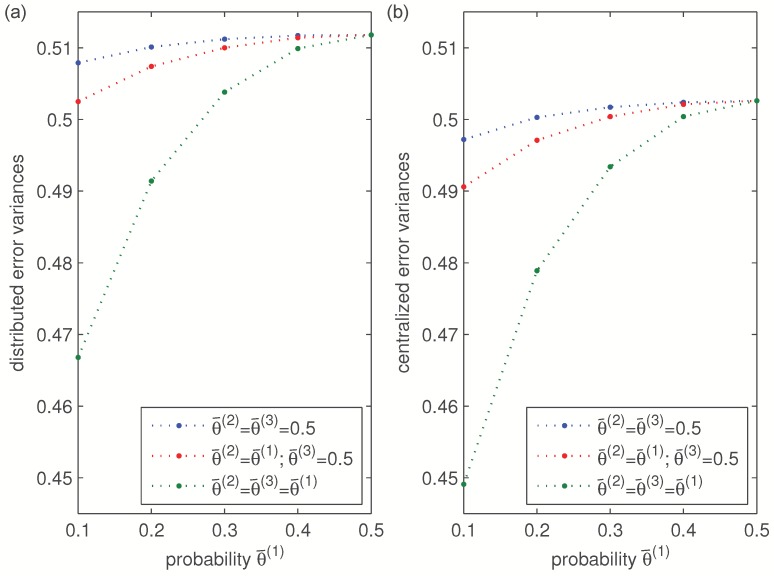
(**a**) Distributed filtering error variances and (**b**) centralized filtering error variances at k=100, *versus* ${\bar{\theta }}^{\left(1\right)}$.

In order to carry out a further discussion on the effects of the sensor random delays, Figure 3 shows a comparison of the filtering error variances in the following cases:

- Case I: error variances *versus* ${\bar{\theta }}^{\left(1\right)}$, when ${\bar{\theta }}^{\left(2\right)}={\bar{\theta }}^{\left(3\right)}=0.5$. In this case, as mentioned above, the values ${\bar{\theta }}^{\left(1\right)}=0.1,\;0.2,\;0.3,\;0.4,\;0.5$, lead to the values ${\bar{\gamma }}^{\left(1\right)}={\bar{\gamma }}^{\left(4\right)}=0.09,\;0.16,\;0.21,\;0.24,\;0.25$, respectively, for the delay probabilities of sensors 1 and 4, whereas the delay probabilities of sensors 2 and 3 are constant and equal to 0.25.
- Case II: error variances *versus* ${\bar{\theta }}^{\left(1\right)}$, when ${\bar{\theta }}^{\left(2\right)}={\bar{\theta }}^{\left(1\right)}$ and ${\bar{\theta }}^{\left(3\right)}=0.5$. Varying ${\bar{\theta }}^{\left(1\right)}$ as in Case I, the delay probabilities of sensors 1, 2 and 4 are equal and take the aforementioned values, whereas the delay probability of sensor 3 is constant and equal to 0.25.
- Case III: error variances *versus* ${\bar{\theta }}^{\left(1\right)}$, when ${\bar{\theta }}^{\left(2\right)}={\bar{\theta }}^{\left(3\right)}={\bar{\theta }}^{\left(1\right)}$. Now, as in Figure 2, the delay probabilities of the four sensors are equal, and they all take the aforementioned values.

Since the behavior of the error variances is analogous in all the iterations, only the results of a specific iteration (k=100) are displayed in Figure 3, which shows that the performance of the distributed and centralized estimators is indeed influenced by the probability ${\bar{\theta }}^{\left(i\right)}$ and, as expected, better estimations are obtained as ${\bar{\theta }}^{\left(i\right)}$ becomes smaller, due to the fact that the delay probabilities, ${\bar{\gamma }}^{\left(i\right)}$, decrease with ${\bar{\theta }}^{\left(i\right)}$. Moreover, this figure shows that the error variances in Case III are less than those of Case II which, in turn, are lower than those of Case I. This is due to the fact that, while the delay probabilities of the four sensors are varied in Case III, only two and three sensors vary their delay probabilities in Cases I and II, respectively. Since the constant delay probabilities of the other sensors are assumed to take their greatest possible value, this figure confirms that the estimation accuracy improves as the delay probabilities decrease.

## 6. Conclusions

In this paper, distributed and centralized fusion filtering algorithms have been designed in multi-sensor systems from measured outputs with random parameter matrices and correlated noises, assuming correlated random delays in transmissions. The main outcomes and results can be summarized as follows:

- *Information on the signal process:* our approach, based on covariance information, does not require the evolution model generating the signal process to design the proposed distributed and centralized filtering algorithms; nonetheless, they are also applicable to the conventional formulation using the state-space model.
- *Signal uncertain measured outputs:* random measurement matrices and cross-correlation between the different sensor noises are considered in the measured outputs, thus providing a unified framework to address different network-induced phenomena, such as missing measurements or sensor gain degradation, along with correlated measurement noises.
- *Random one-step transmission delays:* the fusion estimation problems are addressed assuming random one-step delays in the outputs transmission to the fusion center through the network communication channels; the delays have different characteristics at each sensor and they are assumed to be correlated and cross-correlated at consecutive sampling times. This correlation assumption covers many situations where the common assumption of independent delays is not realistic; for example, networked systems with stand-by sensors for the immediate replacement of a failed unit, thus avoiding the possibility of two successive delayed observations.
- *Fusion filtering algorithms:* firstly, recursive algorithms for the local LS linear signal filters based on the measured output data coming from each sensor have been designed by an innovation approach; the computational procedure of the local algorithms is very simple and suitable for online applications. After that, the matrix-weighted sum that minimizes the mean-squared estimation error is proposed as distributed fusion estimator. Also, using covariance information, a recursive centralized LS linear filtering algorithm, with analogous structure to that of the local algorithms, is proposed. The accuracy of the proposed fusion estimators, obtained under the LS optimality criterion, is measured by the error covariance matrices, which can be calculated offline as they do not depend on the current observed data set.
- *Simulations:* a numerical simulation example has illustrated the usefulness of the proposed algorithms for the estimation of a scalar signal. Error variance comparisons have shown that both distributed and centralized fusion filters outperform the local ones, as well as a slight superiority of the centralized fusion estimators over the distributed ones. The effects of the delays on the estimators performance have been also analyzed by the error variances. This example has also highlighted the applicability of the proposed algorithms to different multi-sensor systems with stochastic uncertainties, which can be dealt with using the observation model with random measurement matrices considered in this paper.

## Figures and Tables

**Table 1:** Sensor 1 data transmission.

Time k	1	2	3	4	5	6	7	8	9	10	11	12	13	14	15
$\theta_{k}^{\left(1\right)}$		1	1	1	0	0	1	0	1	1	1	0	0	1	1
$\gamma_{k}^{\left(1\right)}$		0	0	1	0	0	1	0	0	0	1	0	0	0	
$y_{k}^{\left(1\right)}$	$z_{1}^{\left(1\right)}$	$z_{2}^{\left(1\right)}$	$z_{3}^{\left(1\right)}$	$z_{3}^{\left(1\right)}$	$z_{5}^{\left(1\right)}$	$z_{6}^{\left(1\right)}$	$z_{6}^{\left(1\right)}$	$z_{8}^{\left(1\right)}$	$z_{9}^{\left(1\right)}$	$z_{10}^{\left(1\right)}$	$z_{10}^{\left(1\right)}$	$z_{12}^{\left(1\right)}$	$z_{13}^{\left(1\right)}$	$z_{14}^{\left(1\right)}$	

## Abbreviations

The following abbreviations are used in this manuscript:

LS	Least-Squares
OPL	Orthogonal Projection Lemma
